# Burnout among psychiatry residents in tunisia

**DOI:** 10.1192/j.eurpsy.2022.2201

**Published:** 2022-09-01

**Authors:** M. Abdelkefi, W. Bouattour, N. Bouattour, N. Messedi, F. Charfeddine, L. Aribi, J. Aloulou

**Affiliations:** 1 university hospital center Hedi Chaker Sfax, Psychiatry B, sfax, Tunisia; 2 Hedi Chaker University Hospital, Psychiatry ‘b’ Department, Sfax, Tunisia; 3 Hedi Chaker University Hospital, Psychiatry B, Sfax, Tunisia

**Keywords:** burnout, psychiatry, residents

## Abstract

**Introduction:**

Psychiatry residency training is a stressful transitional period for young doctors who are faced with challenging patients, increased clinical responsibility coupled with lack of clinical experience, and on-call obligations, leaving them at high risk of burnout.

**Objectives:**

To assess the frequency of burnout among psychiatric trainees, and to identify factors associated with severe burnout.

**Methods:**

A cross-sectional study was conducted through an online survey among psychiatry residents working in Tunisian hospitals. Participants completed an anonymous self-administered questionnaire and the Maslach Burnout Inventory (MBI) to assess burnout.

**Results:**

Forty residents completed the survey. The average age was 28.08 ± 2.433. The majority of the participants (87.5%) were females, 27.5% were married and 17.5% had kids. One fifth of the residents were smokers, 22.5 % used alcohol and 5% used cannabis. History of psychiatric disorder was reported by 35% of the participants (depression 15%, anxiety 17.5%, bipolar disorder 2.5%). Half of participants were first year residents and 75% had psychiatry as their first-choice specialty. The majority (72.5%) declared working in poor conditions. Overall, 37.5% of the participants met the criteria for severe burnout. Female gender and poor conditions of the workplace were significantly associated with burnout symptoms (p=0.007 and p=0.014 respectively).

**Conclusions:**

Attention to burnout during residency is important, given the potential to implement preventive and management strategies on time for physicians’ to promote wellness and avoid severe consequences.

**Disclosure:**

No significant relationships.

